# Primordial Germ Cell-Mediated Chimera Technology Produces Viable Pure-Line Houbara Bustard Offspring: Potential for Repopulating an Endangered Species

**DOI:** 10.1371/journal.pone.0015824

**Published:** 2010-12-29

**Authors:** Ulrich Wernery, Chunhai Liu, Vijay Baskar, Zhor Guerineche, Kamal A. Khazanehdari, Shazia Saleem, Jörg Kinne, Renate Wernery, Darren K. Griffin, Il-Kuk Chang

**Affiliations:** 1 Central Veterinary Research Laboratory, Dubai, United Arab Emirates; 2 School of Biosciences, University of Kent, Canterbury, Kent, United Kingdom; Brigham and Women's Hospital, United States of America

## Abstract

**Background:**

The Houbara bustard (*Chlamydotis undulata*) is a wild seasonal breeding bird populating arid sandy semi-desert habitats in North Africa and the Middle East. Its population has declined drastically during the last two decades and it is classified as vulnerable. Captive breeding programmes have, hitherto, been unsuccessful in reviving population numbers and thus radical technological solutions are essential for the long term survival of this species. The purpose of this study was to investigate the use of primordial germ cell-mediated chimera technology to produce viable Houbara bustard offspring.

**Methodology/Principal Findings:**

Embryonic gonadal tissue was dissected from Houbara bustard embryos at eight days post-incubation. Subsequently, Houbara tissue containing gonadal primordial germ cells (gPGCs) was injected into White Leghorn chicken (*Gallus gallus domesticus*) embryos, producing 83/138 surviving male chimeric embryos, of which 35 chimeric roosters reached sexual maturity after 5 months. The incorporation and differentiation of Houbara gPGCs in chimeric chicken testis were assessed by PCR with Houbara-specific primers and 31.3% (5/16) gonads collected from the injected chicken embryos showed the presence of donor Houbara cells. A total of 302 semen samples from 34 chimeric roosters were analyzed and eight were confirmed as germline chimeras. Semen samples from these eight roosters were used to artificially inseminate three female Houbara bustards. Subsequently, 45 Houbara eggs were obtained and incubated, two of which were fertile. One egg hatched as a male live born Houbara; the other was female but died before hatching. Genotyping confirmed that the male chick was a pure-line Houbara derived from a chimeric rooster.

**Conclusion:**

This study demonstrates for the first time that Houbara gPGCs can migrate, differentiate and eventually give rise to functional sperm in the chimeric chicken testis. This approach may provide a promising tool for propagation and conservation of endangered avian species that cannot breed in captivity.

## Introduction

The Houbara bustard is classified as vulnerable on the IUCN Red List and is listed on Appendix I of CITES [1], [2]. It is a medium sized bustard of slender appearance, belonging to the order *Gruiformes* and it is the only species of the genus *Chlamydotis (Chl.)*. The Houbara bustard is separated into three sub-species: *Chl. undulata undulata*, *Chl. undulata macqueenii* and *Chl. undulata fuertaventurae* with *Chl. undulata macqueenii* being the main species in Arabia [3]. Massive captive breeding of Houbara bustards in artificial environments has been attempted, but to date has not been successful in reviving population numbers.

The domesticated chicken (*Gallus gallus domesticus*) belongs to the order *Galliformes*, and can, by contrast, produce fertile eggs non-seasonally throughout year under captive breeding conditions. For this reason, chicken is widely used as and agricultural animal and as an experimental model species. A major priority for Houbara bustard breeding conservationists is to generate a means by which Houbara bustards could be produced with the fecundity of chickens. Primordial germ cell-mediated chimera technology is a promising approach with the potential to achieve this.

Avian primordial germ cells (PGCs), precursor of the germ cells, are epiblastic in origin [4] during early developmental stage that appear in the germinal crescent region [5], [6], until they enter the developing blood vessels in embryonic stage 10–12 [7]. Unlike mammalian PGCs, the avian PGCs migrate through the blood circulation to the developing embryonic gonad, which later develops into the testis or ovary. Circulating PGCs [8], [9] and gonadal PGCs (gPGCs) [10], [11] can be transferred into another chicken embryonic blood circulating system and can contribute a chimeric germ line, which produces functional gametes [12], [13], [14].

Based on intra-species and inter-order chimera technology, derived progenies have been produced by transferring donor cells between Duck (*Anus platrhynchos*) and chicken using blastoderm cell transfer [15] and between Pheasant (*Phasianus colchicus*) and chicken [16]. So far however no viable offspring has been reported from an inter-order chimera as distantly related as Houbara bustard and chicken. The present study was undertaken to provide proof of principle to determine whether Houbara bustard gonadal PGCs can produce functional gametes when in a chicken background.

## Materials and Methods

### Animals

Houbara bustards (*Chlamydotis undulata undulata*) were raised and bred in the Houbara breeding center of the Central Veterinary Research Laboratory (CVRL), Dubai, United Arab Emirates (UAE). Fertilized Houbara bustard eggs were collected after being artificially inseminated during the breeding season. Female Houbara bustards for progeny testing were raised under the same conditions. White Leghorn chickens were maintained in the chicken house of CVRL. Fertilized chicken eggs were collected after artificial insemination (AI). Chimeric chickens were raised under the same conditions.

### Preparation of donor Houbara bustard gonadal cells

Fertilized fresh Houbara bustard eggs were collected and incubated for 8 days at 37.8°C and 60% relative humidity. Blood samples were collected from Houbara bustard embryos to determine the sex before dissecting the gonads, and male embryos were used as gonadal donors. Molecular sexing was performed by PCR as described below. The gonadal tissue was collected individually from Houbara bustard embryos under the stereomicroscope according to [17], and mechanically dissected into small pieces using the tip of 1ml syringes. Dissected tissues were then incubated in Trypsin (0.25%)-EDTA (0.02%) solution (Sigma, T4049) for 7 minutes at 37°C, and dissociated by pipetting with P200 pipetman until there were no obvious tissue clumps observed in Dulbecco's modified Eagle's medium (DMEM) supplemented with 10% Fetal bovine serum (FBS) and antibiotics (L-Glutamine-Penicillin-Streptomycin solution; Sigma, G1146). Cell suspensions were centrifuged at 300g for 5 minutes to remove the supernatant and resuspended in 1ml DMEM (10% FBS). A total of 5µl of cell suspension was taken to analyze the concentration and viability by the Trypan blue exclusion method. The cell concentration was adjusted to 4×10^6^ cells/ml before transfer.

### Transfer of the Houbara bustard gonadal cells into White Leghorn chicken embryos

Fertilized White Leghorn chicken eggs were incubated with the “sharp end up” for 2.5 days until embryonic stage 15–16 [7] at 37.8°C and at 60% relative humidity. A small window (about 10mm in diameter) was made into the shell to expose the embryo on the sharp end. A total of 8×10^3^ cells Houbara bustard gonadal cells in 2 µl DMEM (10% FBS) was injected into dorsal aorta of each chicken embryo with a fine glass pipette. The injected eggs were sealed with a small piece of parafilm and fixed firmly by a heated surgical scalpel. All of the recipient eggs were incubated with the blunt end up until hatch. Injected eggs were candled regularly to check for embryonic growth. Gonadal tissues were collected from the embryos that died during the week before hatch. Houbara bustard cells were detected by PCR with species-specific primers as described below.

### Detection of Houbara bustard PGC-derived sperm from the semen of chimeric roosters

**Figure 1:**
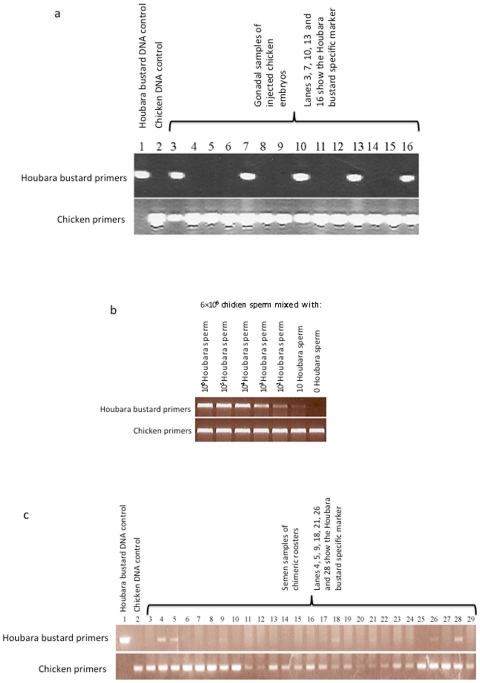
PCR gels with species-specific primers showing the detection of Houbara bustard DNA. (**a**) Detection of bustard DNA in the gonadal tissue of chimeric chicken embryos. Lanes 3, 7, 10, 13, and 16 show the bustard specific DNA; (**b**) Species identification sensitivity test: 6×10^6^ chicken sperm mixed with decreasing quantities of bustard sperm; (**c**) Detection of bustard DNA in the semen of chimeric roosters. Lanes 4, 5, 9, 18, 21, 26 and 28 show the bustard DNA.

Chimeric chickens were raised to sexual maturity. Semen samples were collected from 34 chimeras weekly for a period of three months. Freshly collected semen was diluted 20 times in calcium and magnesium-free phosphate buffered saline (PBS) and 50 µl of diluted semen was used to detect the existence of Houbara bustard sperm by a PCR species identification test as described below. The sensitivity of the PCR species identification test was determined with mixture of samples from Houbara bustard and chicken sperm with a graded ratio from one to 10 million sperm (Table 1, Figure 1b).

**Table 1:** Detection of donor cell-derived Houbara bustard sperm in the semen of chimeric roosters.

Birds ID	The number of samples collected	The number of positive samples	Percentage
hw329	12	1	8.3%
hw335	12	1	8.3%
hw336	10	1	10%
hw345	14	2	14.3%
hw386	12	1	8.3%
hw390	13	1	7.7%
hw439	9	1	11.1%
hw473	13	1	7.7%
**Total**	**95**	**9**	**9.5%**

### Progeny test

During the breeding season between January and May, semen samples were collected from 8 male chimeric roosters that had produced Houbara-DNA positive samples previously. Fresh samples were re-checked by PCR for presence of Houbara bustard DNA. Doses of 0.3–0.5ml of these semen samples were artificially inseminated twice a week into three virgin female Houbara bustards. The resulting eggs were collected and incubated until hatch under the conditions as described above. The remaining unhatched eggs were opened after 25 days incubation to examine the fertility and development.

Blood was collected from the resulting progeny; a piece of muscle tissue was dissected from the body of dead embryos. Sex determination, species identification, and genotyping and parentage verification tests were conducted with these samples by molecular analysis as described below (Table 2).

**Table 2:** Progeny test of germline chimeric roosters by artificial insemination with female Houbara bustards.

	Female Houbara ID	Total times of AI	The number of positive samples inseminated	The number of eggs	Fertility	Hatchability
1^st^ season	000149	36	4	7	0	0
	020154	36	5	8	0	0
	010242	36	5	5	0	0
2^nd^ season	000149	27	5	12	0	0
	010242	27	4	2	0	0
3^rd^ season	000149	18	6	1	0	0
	020154	18	6	10	20.0% (2/10)	50.0% (1/2)
Total		198	35	45	4.4%	2.2%

### Molecular analysis

#### DNA Extraction

Pretreatment was done according to the sample type. a) Whole blood; 50µl of blood, collected in EDTA-vacutainer tubes, was mixed with 20µl of 10mg/ml Proteinase K and 500µl of tissue lysis buffer, incubated at 56°C for 2 hrs. b) Tissue samples: about 25mg of the tissue was mixed with 20µl of 10mg/ml Proteinase K and 200µl of tissue lysis buffer, incubated at 56°C for 2 hrs. c) Semen samples: 25–50µl of semen was treated with 25µl of 0.2M NaOH and incubated at 95°C for 10 min, and followed by neutralization using 0.25M Tris-HCl (pH 8). Subsequently, any of the above lysed cells were mixed with an equal volume of Phenol ∶ Chloroform ∶ Isoamylalcohol (25∶24∶1). The DNA was precipitated using 1/10 volume of 3M sodium acetate and 2.5 volumes of absolute ethanol. The DNA was dissolved in about 200ul of TE buffer (pH8), quantified by Nanodrop ND-1000 Spectrophotometer V3.5 (Nanodrop, Technologies Inc, USA), and adjusted to about 50ng/µl for each PCR reaction.

#### Species Identification

The specific primers CHN1F and CHN1R, BTD2F and BTD2R (Table 3) were used for identification of chicken and Houbara bustard species, respectively. Chicken primers were designed using the sequence on chromosome 11 at the NCBI database, Trace/Gallus_gallus_WGS [18]. The primers for Houbara species identification are designed from the cytochrome b gene found in the mitochondria [19]. The Gene Bank accession number for this sequence is AF077932. The sequence of primers, expected product size and references are given in Table 3. Amplification was performed according to the following cycling conditions: initial denaturation at 95°C for 5 min, followed by 40 cycles of 94°C for 45sec, 58°C for 30sec and 72°C for 45 sec. The final extension was carried out at 72°C for 5 min.

**Table 3:** Primer list for species identification test and molecular sexing.

No.	Primer	Primer sequence (5′-3′)	Tm (°C)	Product Size (bp)	Method
1	CHN1F	CCT CCC AGT CCC AGT AAG AAG TAG	58	221	Species Identification [18]
	CHNIR	CAA CAT GAT GGG CGA GTG CT			
2	BTD2F	GCC TCC GTA CTA GTC CTG TTC TTA	58	315	Species Identification [19]
	BTD2R	GGG TGT AGT CTT CAT TCT TTG GTT			
3	USP1	CTA TGC CTA CCA C(A/C)T TCC TAT TTG C	58	380	Bird Sexing [20]
	USP3	AGC TGG A(T/C)T TCA G(A/T)(C/G) CAT CTT CT			
4	MYO INT1	AGC CCT GGA GGA TCC ATT GG	58	200	Bird Sexing [20]
	MYO INT2	CAG TGA GGT CTA GTA TGC AAG G			
5	P3	AGA TAT TCC GGA TCT GAT AGT GA	55	110	Bird Sexing [21]
	P2	TCT GCA TCG CTA AAT CCT TT			

#### Sex Determination

Sex identification was performed according to a) [20]; primers USP1 and USP3 were used to determine the sex, Myo INT 1 and Myo INT2 were used as internal control primers (Table 3). The cycling conditions were as follows: an initial denaturation at 95°C for 5 min was followed by 30 cycles of denaturation at 95°C for 30sec, annealing at 50°C for 30 sec and extension at 72°C for 1 min and a final extension at 72°C for 10 min. b) primers P2 and P3 [21] were used to amplify a 110bp fragment that was restriction digested using the enzyme HaeIII. The thermal cycling conditions included a 5 min initial denaturation at 95°C followed by 40 cycles of 95°C 30sec, 55°C for 15 seconds and 72°C for 15 seconds. This was followed by a 1 minute annealing at 56°C and a final extension at 72°C for 5 minutes. Part of the amplified PCR product was digested with HaeIII at 37°C for 1 hour. The digested and the undigested products were run parallel on a 4% agarose gel to determine the sex and make sure all the samples had been amplified.

#### Microsatellite analysis

The genome DNA samples were extracted from blood of the live progeny and related adult birds, and subjected to microsatellite analysis for genotyping and parentage verification. The list of primers, their sequences and references are shown in Table 4. PCR was performed in a 12µl reaction volume using 10× Fast Start Taq buffer and Fast Start Taq Polymerase (Roche Diagnostics, USA). M13 F or R tailed primers were used and grouped into 3–4 multiplexes. The amplified products were analyzed by running on ABI 3730 XL DNA Analyzer and the genotypes were analyzed using the Genemapper V4.0 (Applied Biosystems Inc. USA).

**Table 4:** Primer list of genotyping analysis using Houbara bustard microsatellites (STR) markers [26].

No.	Primer name	Primer sequence
1	BusA2F-MR	CAG GAA ACA GCT ATG ACC GCA GCA AAG AGA AGC AAA G
	BusA2-R	CAA GCT CCT GTA GGG ATC A
2	BusA10F-MR	CAG GAA ACA GCT ATG ACC GCT GAA TCT TGG CTT AGA TG
	BusA10-R	AAG GAA CAG AAA GGT TCT CTG
3	Bus A18F-MF	TGT AAA ACG ACG GCC AGT CTG GCA TTT CAG TGG CTT C
	Bus A18R	CCC AGG GCA GAA CAG ATC
4	Bus A22F-MF	TGT AAA ACG ACG GCC AGT ACA CGT ATG CAC GCA CAT C
	Bus A22R	TGC AAG GGG TTA ATG CTG T
5	BusA29F-MR	CAG GAA ACA GCT ATG ACC GAG AGG GAA AGA CAC ACG TA
	BusA29-R	AAA TTG CTG GAG AGT CAG G
6	BusA120F-MR	CAG GAA ACA GCT ATG ACC GGA GGA GAA TGC AGC AGG T
	BusA120R	GCA TTA AGA TGC ACC CAC AA
7	BusA204F-MF	TGT AAA ACG ACG GCC AGT GCA TTT CAG TGG CTT CTC C
	BusA204R	TTT GCT GGT GCC AGA GTC
8	BusA205F-MF	TGT AAA ACG ACG GCC AGT GCT ACG ATA CAA AAC CAA AAC T
	BusA205R	CAT GCA ATG TGG AGT GAC T
9	BusA210F-MF	TGT AAA ACG ACG GCC AGT CTC CAT TTT CAA CCA ATC TTC
	BusA210R	GCG CTC TTT TAA TAG GTC AAA
10	BusD110F-MF	TGT AAA ACG ACG GCC AGT CCA GCC TAA AGG ATG TGA A
	BusD110R	TGA TGA AAT GGC AGA TAG ATG
11	BusD117F-MF	TGT AAA ACG ACG GCC AGT GCT CGT GAA ACC AGT GTG
	BusD117R	GCC AGA CAG AAA CAG AAG G
12	BusD118F-MR	CAG GAA ACA GCT ATG ACC AGA AAC CTG GGG TGA TGA
	BusD118R	AAT CCC TAC CTC TTC CCT G
13	BusD119F-MR	CAG GAA ACA GCT ATG ACC ACT CAG CTC TGG GGA AGT TAT G
	BusD119R	TTC TCT TTG TGG ATC CTC AAT G

## Results

### Harvest of donor Houbara bustard gonadal cells

Chicken embryos developed to embryonic stages 26, 28 and 30 after 5, 6 and 7 days incubation, while Houbara bustard embryos appeared to have comparatively slower development in the same age, approximately 1–2 days delay, and showed significant individual variance as well which is in line with its varied whole incubation period (22–24 days). The total number of gonadal cells in 8dpi Houbara bustard embryos was 102.7±21.2×10^3^ cells (n = 15) with a viability of 96.5±1.6% in female embryos, and 114.8±20.5×10^3^ cells (n = 23) and 96.9±1.2% in male embryos.

The morphological characteristics of Houbara bustard gPGCs were similar to that of chicken. They can easily be distinguished from somatic cells by being rounder and larger in size (12–15 µm in diameter) as well as richer in granules in the cytoplasm than somatic cells.

### Production of chimeric chickens

In total, 138 chicken embryos were injected with Houbara bustard gonadal cells from individual male embryos, out of which 83 survived and hatched after 21 days incubation with a hatchability of 60.1% (83/138). All of the hatched putative chimeric chicks had a typical White Leghorn phenotype. The chimeric chicks were raised under normal conditions, and 35 male and 35 female birds reached to sexual maturity after 5 months. Houbara bustard-specific DNA fragments were amplified from 31.3% (5/16) gonadal tissues of the injected chicken embryos pre-hatch (Figure 1a). These results suggested that the transferred Houbara bustard cells had colonized and survived in chimeric chicken gonads, even across the considerable phylogenetic distance.

### Molecular analysis of chimeric embryos and semen samples from adult chimeric roosters

Houbara bustard species-specific primers have been developed and used to detect Houbara bustard sperm produced by chimeric chicken. The sensitivity of species identification PCR test was determined with the mixed samples of Houbara bustard and chicken sperm. Certain graded number (from one to 1 million sperm) of Houbara bustard sperm was mixed with six million chicken sperm. Results showed that Houbara bustard sperm could be detected from the mixture semen as low as 10 Houbara bustard sperm in chicken semen containing 6 million sperm (Figure 1b).

A total of 302 semen samples were collected from 34 chimeric roosters. Houbara bustard species-specific DNA fragments were amplified from the semen samples of 23.5% (8/34) birds. Since the semen does not contain somatic cells, these results indicated that eight chimeric roosters were capable of producing Houbara bustard sperm and therefore considered as germline chimeras. Further 95 semen samples were collected from these 8 germline chimeras, out of which 9.5% (9/95) were confirmed as Houbara bustard-DNA positive (Figure 1c; Table 1). These results suggested that Houbara bustard gonadal cells injected in the early embryonic stage might be able to differentiate into sperms in the testis of chimeric rooster.

### Progeny test

**Figure 2:**
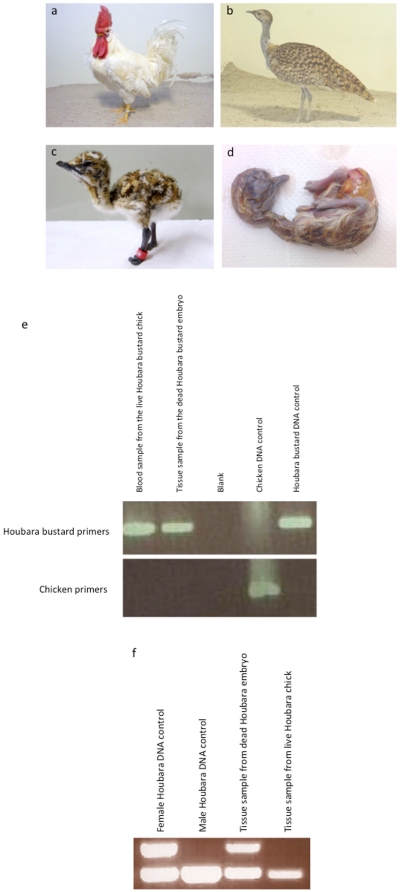
Parents, offspring and progeny tests. (**a**) Germline chimeric rooster; (**b**) Female Houbara bustard (HB020154); (**c**) Houbara bustard chick generated from donor cells derived from Houbara bustard sperm produced by chimeric rooster (**d**) Dead Houbara bustard embryo from chimeric rooster; (**e**) species identification test by PCR; (**f**) molecular sexing by PCR of the dead Houbara embryo and the live Houbara chick.

Three female Houbara bustards were artificially inseminated 198 times with the semen samples collected from these 8 germline chimeric roosters. A total of 23 samples inseminated were confirmed containing Houbara bustard sperms by molecular analysis. Subsequently, a total of 45 Houbara bustard eggs were obtained and incubated, of which two eggs were found to be fertile. One successfully hatched after 22 days of incubation, while the other fully developed embryo died before hatching (Figure 2a–d, Table 2). The chick and the dead embryo showed typical Houbara bustard phenotype, albeit with minor deformity in the toes of both feet.

### Species identification and parentage test of the resulting progenies

A Houbara bustard species-specific DNA fragment was amplified from both offspring, however chicken species-specific DNA fragment was not amplified (Figure 2e) providing strong evidence that the resulting offspring were genetically pure Houbara bustard. The hatched chick was identified by PCR as male; the dead embryo was female (Figure 2f). The genotyping analysis using Houbara bustard and chicken microsatellites (STR) markers also provided further support for this (Table 5). Firstly, it provided independent confirmation that the resulting offspring were genetically pure Houbara bustard, not chicken. Secondly, it illustrated that the offspring were not produced through parthenogenesis, as their genotypes are not fully derived from their mother. Moreover, it verified the parentage as the comparison of offspring genotypes with the parental (donor) genotype. Unfortunately no sample was kept from the donor animal and thus the potential donor's genotype was deduced from its parents. At the same time one can predict the donor's genotype from its offspring and their mother. By comparing these two derived genotypes, we predicted the donor's genotype as described in Table 5.

**Table 5:** Genotyping analysis of the Houbara bustard family using Houbara bustard microsatellites (STR) markers [26].

No.	Markers	Mother Houbara	Live Houbara chick	Dead Houbara embryo	Predicted donor	Grand mother	Grand father
1	BusA2	155/165	155/159*	155	159/155	159/165	155/159
2	Bus A10	162	162	162	162	162	162
3	BusA18	144/148	148/146*	144/146*	146/?	146/148	146
4	BusA22	158	158	158	158	158	158
5	Bus A29	158/164	158	164/160*	158/160	160/164	158/160
6	Bus A120	282/288	282/280*	282/280*	280/?	280/290	280
7	Bus A204	176/180	180/178*	176/178*	178	178/180	178
8	Bus A205	216	216	216	216	216	216
9	Bus A210	146/150	146/142*	150/142*	142/?	150/152	142/146
10	Bus D110	161/165	161	161/153*	153/161	161/165	161/153
11	BusD117	168/172	172	168/172	172/176	176	172
12	Bus D118	274/278	274	274/266*	266/274	274/278	274/266
13	BusD119	260/268	260	260	260/268	268	260

*alleles from paternal genome.

## Discussion

In the present study, a viable Houbara bustard was successfully hatched between a male chimeric rooster and pure female Houbara bustard for the first time. The study demonstrates that PGCs can be harvested from embryos with a high viability and that germ cells are able to migrate into the chicken recipient gonad, survive and differentiate into functional sperm alongside the endogenous chicken sperm. In other words, the male chicken reproduction system can support Houbara bustard spermatogonial development in the testis. The chimeric rooster thus served as a surrogate father of the chick, perhaps suggesting that the reproduction organs, including hormonal systems, might be widely conserved in different avian species and orders. This might help us to understand the wild bird reproduction system particularly the male spermatogenesis process.

Already pheasant PGCs derived progenies were successfully produced from chimeric roosters [16]. Also donor cells were detected in semen samples from chicken-quail chimera; however, no progeny was obtained [22]. Highly sensitive molecular sexing and species identification PCR methods for Houbara bustard cells were developed as a result of this research, also providing strong molecular tools for Houbara bustard research, including the tracing the Houbara bustard cells or sperm, and the tracing of the donor cells in the chimeric embryo or rooster.

The Houbara bustard reaches sexual maturity at about two years (one year at the earliest) [23]; this was confirmed in our breeding center. In contrast, chicken reaches sexual maturity in about five months. In the present study, it was confirmed that Houbara bustard sperm could be detected when the chimeric rooster first reach sexual maturity, indicating that donor germ cell differentiation occurs in same time that the recipient spermatozoa forms. In other words, the environment of the host chimeric rooster gonads supports the development of host germ cell as well as of the donor germ cells. The donor Houbara bustard DNA was not detected in every weekly semen sample, but no specific pattern was observed. However, donor DNA was detectable from the host semen for up to three years. It is not clear whether or not the negative result on donor sperm in the host semen is due to the PCR sensitivity. Furthermore, Houbara bustard DNA was detected in semen during off- breeding season. It is also not clear if the donor sperm was generated and released in the host testis by its own spermatogenesis wave. The spatio-temporal mechanism of the Houbara bustard spermatogenesis in the host seminiferous tubules needs to be investigated further. Unlike the seasonal breeding Houbara bustard germ cells differentiation pattern, these observations suggest that the Houbara bustard PGCs follow the non-seasonal breeding chicken reproduction pattern. This will increase the chance to produce even more Houbara bustards using the domestic male chicken reproduction system all year round.

The efficiency of the progeny production is still very low, only one live chick and one dead before hatching. It is important to note however that both were identified as pure Houbara bustard, not hybrid, and not from a parthenogenic development. Furthermore we provided strong evidence that the chick and embryo were the progeny of the chimeric rooster and the Houbara bustard by the parentage test, which is a strong molecular tool, for the first time. It is reasonable to assume that there is some kind of competition between the donor and recipient germ cells in the chimeric body, but still it is not well understood why the number of Houbara bustard sperm in the chimeric rooster semen is fluctuating. To increase the number of donor derived sperm few efforts need to be made in the future, such as 1) increasing the donor PGC by purification and culture in vitro [24], [25], 2) reduce the endogenous chicken PGCs using mechanic or chemical methods [26], 3) finding the better transfer timing.

Furthermore, production of female chimeras between chicken and other domestic avian species by PGCs transfer has also been reported. However, female donor PGCs derived offspring has not been achieved from the chimeric hens [27]. If female PGCs could differentiate into functional ova in the ovary of chimeric hen, Houbara bustards could be reproduced through male and female chimeric chicken. This will greatly increase the Houbara bustard population in captivity, take away the pressure from hunting wild birds, and discourage people from trading or smuggling Houbara bustards. In the end, this technology could also be applied to the conservation of other endangered avian species that cannot be bred in captivity.
